# The physicochemical properties of the spirulina‐wheat germ‐enriched high‐protein functional beverage based on pear‐cantaloupe juice

**DOI:** 10.1002/fsn3.2963

**Published:** 2022-07-22

**Authors:** Hamed Hassanzadeh, Babak Ghanbarzadeh, Yaseen Galali, Hamed Bagheri

**Affiliations:** ^1^ Department of Food Science and Hygiene, Faculty of Para‐Veterinary Ilam University Ilam Iran; ^2^ Department of Food Science and Technology, Faculty of Agriculture University of Tabriz Tabriz Iran; ^3^ Department of Food Engineering, Faculty of Engineering Near East University Mersin Turkey; ^4^ Food Technology Department, College of Agricultural Engineering Sciences Salahaddin University‐Erbil Erbil Iraq; ^5^ Department of Nutrition and Dietetics Cihan University‐Erbil Erbil Iraq; ^6^ Department of Research and Development Takdaneh Co. Marand Iran

**Keywords:** antioxidant capacity, functional juice, spirulina, total phenol, wheat germ powder

## Abstract

The formulation of a novel functional juice, enriched with wheat germ powder and spirulina algae and based on cantaloupe and pear juice, was optimized by D‐optimal combined design. Firstly, sensory evaluation was performed by hedonic test to evaluate the organoleptic properties, and organoleptically desirable samples were screened for further experiments. Various chemical experiments including PH, acidity, formalin index, total phenol, flavonoids, antioxidant capacity, mineral contents (Fe, Zn, Ca, P, K, Mg, and Cu), and fatty acids profile were evaluated. The steady shear flow rheological test also was performed on the screened samples. The results of sensory evaluation showed that the samples containing 1% spirulina and wheat germ had the highest organoleptic score. The results of physicochemical tests on the selected samples showed that the addition of spirulina and wheat germ powder had little effect on pH, acidity, and formalin index but they affected brix, dry matter, and protein content. Also, the addition of spirulina and wheat germ powder, changed the amounts of antioxidant capacity (from 90 to 98%), total phenol (from 4 to 22 mg GAE/g), and flavonoid content (from 5 to 15 mg/L) in the functional beverages. Furthermore, the results of rheological tests showed that the addition of wheat germ powder in the functional fruit juices increased apparent viscosity however; spirulina did not affect important change in rheological properties. The GC‐Mass analysis presented fatty acid profiles of the functional beverages and confirmed the presence of polyunsaturated fatty acids (for example decanoic acid and heptadecanoic acid) in the samples.

## Graphical abstract

The formulation of a novel functional juice enriched with wheat germ powder and spirulina algae based on cantaloupe and pear juice was optimized by D‐optimal combined design. Various chemical experiments including PH, acidity, formalin index, total phenol, flavonoids, antioxidant capacity, mineral contents (Fe, Zn, Ca, P, K, Mg, and Cu), and fatty acids profile were evaluated.

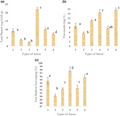

## 1 INTRODUCTION

Functional foods are foods that have proven benefits for the maintenance or promotion of health beyond basic nutrition and help reduce the risk of disease. Functional foods include conventional foods, modified foods (i.e., fortified, enriched, or enhanced), medical foods, and foods for special dietary use. Functional beverages are nonalcoholic drink containing health‐promoting ingredients like minerals, vitamins, amino acids, dietary fibers, probiotics, added raw fruits and herbs, polyphenols, pigments, etc., which positively affecting one or more target functions in the body (Rojo‐Poveda et al., 2019). Algae are a diverse group of macroscopic and microscopic organisms ranging from the prokaryotic Cyanophyta (blue‐green algae) to the eukaryotic algae, from unicells (microalgae) to multicellular organisms (Villarruel‐López et al., 2017). Microalgae are source of proteins with high biological value which has potential to meet the dietary requirements of a growing population. In addition, it is a source of protein, the presence of various bioactive components in microalgae provides several health benefits effects (Koyande et al., 2019).

*Spirulina platensis* blue‐green microalgae are well known due to their unique nutritional quality. This microalga contains high protein (60%–70% dry weight), low‐fat, high amounts of vitamins, especially vitamin B12, iron, phycocyanin pigments, and essential fatty acid, and could be used in food as functional ingredient with high nutritional and health‐promoting value. (Mostolizadeh et al., 2020).

Wheat germ is a precious by‐product derived from the milling industry and is rich in protein with high essential amino acids (lysine, methionine, and threonine), fatty acids, minerals, vitamins, tocopherols, and phytosterols, and hence, wheat germ seems a valuable ingredient for enrichment of functional food and beverage and development of healthy food (Boukid et al., 2018; Çalışkan Koç & Özçıra, 2019).

In recent years, some research works have been reported on development of function foods such as microalgae‐based sourdough (Niccolai et al., 2019), lemon juice enriched with resveratrol‐γ‐cyclodextrin (Silva et al., 2021), pineapple juice containing ginger turmeric extract (Ogori et al., 2021), pineapple and orange juice enriched with hibiscus sabdariffa extracts (Ogundele et al., 2016). The distinguishing feature and novelty of this research is the simultaneous use of two nonanimal protein sources (spirulina and wheat germ) with unique and well‐known nutritional properties (enrich in essential fatty acids, essential amino acids, minerals, pigments, and antioxidants) to formulate a functional protein drink (pear‐cantaloupe) that can be useful for all age groups, especially children and the elderly. The present study aimed to develop of a type of protein‐enriched functional beverage with adding of nonanimal protein sources (spirulina and wheat germ) in pear‐cantaloupe juice. These protein sources also are enrich of essential fatty acids, essential amino acids, minerals, pigments, and antioxidants.

## 2 MATERIALS AND METHODS

### 2.1 Preparation of juices

In this research, first, according to the experimental design prepared by Design‐Expert software, juices were prepared based on 50% natural juice and after controlling the quality characteristics of the juice, including its acidity, the samples were poured into cans and transferred to the production line. The cans were sealed and pasteurized in a pasteurized tunnel and then cooled. Finally, the physicochemical properties of the samples began as follows:

### 2.2 PH, brix, titratable acidity, and formalin index measurement

Total soluble solids and pH measurement total soluble solids (°Brix) were measured with a refractometer (Atago, Minato‐ku, Japan) and pH was measured using a pH meter.

For determination of titratable acidity, a known volume of each sample was placed in a 250 ml beaker and 50 ml of distilled water was added. Further, this solution was titrated against standardized 0.1 N NaOH to the phenolphthalein endpoint (pH 8.2 ± 0.1). The volume of NaOH was converted to grams of citric acid per 100 ml of juice (% TA) based on the method of Sadler and Murphy, (2010) and the total acidity was calculated using equation (1): 100 *Vs* (1) where *V* is titer volume of NaOH and *Vs* is the volume of the strawberry juice sample.

Formalin index (g 100/g juice), total sugars, and reducing sugars in the fruit juices were determined according to Iran's Standard based on Lane‐Inon measurement (Ghasemi et al., 2019).

### 2.3 Antioxidant capacity measurement

An ethanolic DPPH solution (100 mM) was used for determinations. Ethanol (0.1 ml) was mixed with 3.9 ml of DPPH (100 mM) to determine the initial absorbance of the DPPH solution. Next, 0.1 ml of sample extract was added to 3.9 ml of 100 mM DPPH solution. The mixture was shaken immediately and allowed to stand at ambient temperature in the dark. The decrease in absorbance at 517 nm was measured after 60 min. The radical scavenging activity was expressed as the inhibition percentage of the DPPH radical (Hassanzadeh et al., 2019). The free radical scavenging ability of the beverage samples against DPPH (1,1‐diphenyl‐2‐picrylhydrazyl) free radical was evaluated. The DPPH free radical scavenging ability was subsequently calculated (Bahrami et al., 2017Hassanzadeh et al., 2022).

$$\begin{array}{c} \%\text{Inhibition}=\left[\left(\text{Absorbance of control}-\text{Absorbance of the test sample}\right)/\text{Absorbance of control}\right]\times 100 \end{array}$$

### 2.4 Total phenolic content (TPC) determination

TPC was determined spectrophotometrically using the Foline–Ciocalteu reagent (FCR) according to the methodology proposed by Viacava and Roura (Viacava et al., 2015) with modifications. Some diluted samples were added to 1000 ml of FCR (diluted 1/10). After 3 min of incubation at ambient temperature, 800 ml of 7.5% Na2CO3 solution was added and the reaction mixture was incubated for 2 h at the same temperature and in a dark place. The absorbance was measured at 765 nm using a UV–Vis spectrophotometer (1601 PC UV–visible, Shimadzu Corporation) and TPC was calculated using gallic acid as standard. Results were expressed as mg gallic acid equivalents (mg GAE)/100 ml of juice (Cassani et al., 2016).

### 2.5 Total flavonoid content determination

The sample (250 μl of 1:10 dilution of a beverage sample or 250 μl of 1 mg/ml grape extract in 30% methanol) was mixed with 1.25 ml distilled water and 75 μl of 5%NaNO2 (w/w). After 5 min, 150 μl of 10% AlCl3 (w/w) was added and allowed to react for 6 min. Then, 500 μl of 1 M NaOH was added. The final volume was adjusted to 3 ml with distilled water. The mixture was mixed well and the absorbance was immediately measured at 510 nm against the blank using UV–Visible spectrophotometer ((UV1601, Shimadzu Scientific Instruments [Oceania] Pty. Ltd.)). Catechin (10–750 μg/ml) was used to plot a standard curve. Total flavonoid contents in all samples were expressed as the mean ((milligrams of catechin equivalents [CE] per 100 ml of beverage or gram extract)) ± SD for triplicate results of analysis (Nanasombat et al., 2015).

### 2.6 Analysis of fatty acid composition

Methyl esters of fatty acids were prepared in accordance with the method of Morrison and Smith (1964) with some modifications. For sample 100 mg adding 1 ml BF3/methanol (14%) and 1 ml hexane. The tube is vortexed and placed under nitrogen for 60 min at 100°C. Esters of fatty acids were then extracted by adding 1 ml of hexane and washing with 2 ml of distilled water. After the centrifugation step (2500 *g*, 10 min, 20°C), the supernatant is recovered in vials and then injected into the GC column. Methyl esters were analyzed by GC‐type CG‐2010 Plus, Shimadzu, equipped with a flame ionization detector and a capillary column of 60 m in length, 0.25 mm internal diameter, the thickness of the film is 0.20 microns. The oven temperature is 200°C. The detector and the injector are at a temperature of 250°C. The samples were separated on the column using helium as the carrier gas with a flow rate of 0.8 ml/min. The sample is injected in split mode. The temperature program used in the analysis is to keep the unit at 120°C for 2 min and then climb to 180°C for 2 min and keep the sample at 220°C for 25 min. The peak integration is done on the software GC, GC solution (Shimadzu). Peak identification of fatty acids on the chromatogram is made using standard fatty acids (Restek, Food industry FAME Mix—methylene chloride 30 mg/ml) (Mahmoud et al., 2015).

### 2.7 Mineral content determination

In the present study, the wet digestion method has been used. Five milliliters (approx) of well‐mixed juice sample was taken and transferred to a 100 ml beaker and 5 ml on Nitric acid (65%) was added and covered with watch glass and then the mixture was heated to 95°C on a hot plate, and finally the digestion continued till no brown fumes evolved and the solution becomes clear and colorless. The beaker is then cool to room temperature. The solution is then transferred into a volumetric flask and made up the mark with double distilled water. A known weight of the sample is digested using a wet digester with concentrated nitric acid. The digested sample is diluted to the known volume and analyzed in ICP‐MS/AAS. Atomic Absorption Spectroscopy coupled with inductively coupled plasma Mass spectrometry was used in the study, instrumental conditions were adjusted, instruments were calibrated by blank solution and finally, metals/minerals contents of the fruits were analyzed. Cu, Zn, As, Cd Hg, and Pb were analyzed by ICP‐MS while Ca, Fe, Na, and K were analyzed by AAS. (Cassani et al., 2016).

### 2.8 Steady‐state shear rheology

Steady‐state shear rheology tests were carried out using a Physica MCR301 Rheometer (Anton Paar, Germany) equipped with a Couette flow measuring cell (Ref. DG27/T2000/SS). Sample temperature was achieved at 20 ± 0.1°C using a Peltier system and a fluid circulator Viscotherm VT 2 controlled directly from the Physica MCR. After 5 min of thermal stabilization, each 11 ml sample was submitted to a flow test in the 10–400/s shear rates range (Laux et al., 2013).

### 2.9 Sensory evaluation

Sensory evaluation was carried out by a hedonic scale consisting of 10 points (1–10), where 9–10 excellent, 7–8 very good, 5–6 good, 3–4 fair, and 1–2 poor. An internal panel of 15 members evaluated the products for color, appearance, taste/flavor, mouthfeel, and overall acceptability (Renuka et al., 2009).

### 2.10 Microbiological quality

The fresh, as well as stored samples of nectar were analyzed for their microbial load using standard methods as described by Binduheva and Negi (2014). Nectar (1 ml) was inoculated onto Plate Count Agar and Potato Dextrose Agar for analyzing its total bacterial counts, and yeast & mold counts, respectively; and the presence/absence of Escherichia coli was checked by plating on HiChrome E. coli Agar using pour plate technique. The colonies developed in Plate Count Agar (24 h at 37°C), and Potato Dextrose Agar plates (25°C for 2–5 days) were counted and expressed as log CFU/ml. The HiChrome E. coli Agar plates were incubated for 48 h at 37°C and the presence/absence of typical E. coli colonies was noted.

### 2.11 Experimental design and statistical analysis

In this research, the Box–Behnken design has been used in Design‐Expert software. Each of the treatments (spirulina algae and wheat germ powder) was used in combination and individually in different ratios and then by selecting the appropriate statistical model in the same software (Table 1), statistical analysis was performed at (*α* = 0.05) and the optimal samples were selected.

**TABLE 1:** Experimental design (combined design) used for optimizing organoleptic characteristics

Run	Component 1 A:Spirulina	Component 2 B:Wheat germ powder	Factor 3 C:Protein %
1	100	0	1
2	0	100	3
3	75	25	1
4	0	100	1
5	100	0	2
6	25	75	1
7	25	75	1
8	0	100	1
9	25	75	3
10	75	25	3
11	0	100	2
12	100	0	3
13	100	0	3
14	66.6	33.3	2.5
15	50	50	2
16	66.6	33.3	1.5
17	25	75	3

Table 1 lists the different formulations prepared. As shown in Table 1, the total protein content of the juice is adjusted from 1 to 3% of the total weight of the juice based on spirulina and wheat germ powder (in their various proportions). Then, the optimal samples were compared in a completely randomized design in Minitab software, and mean comparisons were performed by multiple range Duncan test.

## 3 RESULT AND DISCUSSION

### 3.1 Measurement of physicochemical properties of raw materials

The amount of ash, moisture, protein, acidity, and fat for the raw materials including wheat germ powder and spirulina algae were measured and the values shown in Table 2 were obtained.

**TABLE 2:** Physicochemical properties of raw material

Types of tests	Types of materials	Spirulina	Wheat germ powder
Ash (%)		0.85 ± 0.1	1.22 ± 0.09
Humidity (%)		12 ± 1.1	17 ± 1.2
Protein (%)		65 ± 2.3	18.15 ± 1.41
Fat (%)		0.15 ± 0.02	9.67 ± 0.8
Acidity (%)		‐	1.8 ± 0.11

### 3.2 Sensory evaluation for screening

Considering that one of the most important criteria for producing a new product and presenting it to the market is the discussion of the customer‐friendliness of that product. In this project, first about 15 employees, as panelist, to rate the samples prepared in terms of perfume, taste, color, and general acceptance, were selected. Evaluators scored from 1 to 10 for very weak to excellent samples, respectively, and the scores were transferred to the software and the optimal sample was selected organoleptically.

The results of the sensory evaluation showed that samples containing 1% protein (total spirulina and wheat germ) had the highest organoleptic score. Also, sensory evaluation during the storage period of the product showed that the samples with higher amounts of spirulina had an unpleasant taste and only the samples containing very low amounts of spirulina could be consumed. It should be noted that the pasteurization heat also caused the product to taste bad in samples containing higher amounts of spirulina.

Contradictory results have been reported regarding the effect of adding spirulina on the sensory properties of food. For example, Niccolai et al. (2019) on the effect of adding spirulina on the sensory properties of “crostini” (a leavened bakery product mostly consumed in Italy and Europe) stated that no significant effect was found by addition of spirulina, regarding taste, smell or texture attributes, while color was significantly has dropped. Some researchers explained the results of sensory evaluation in microalgae‐based food products such as bread (Saharan & Jood, 2017), pancakes (Kumar, 2017), croissants (Massoud et al., 2016), cookies (Batista et al., 2017), and pasta (Fradique et al., 2013) finding that these products showed different satisfactory in terms of total acceptance. Egea et al. (2014) found that the addition of A. *platensis* (spirulina) biomass (2% and 5%) in the cookies decreased the total acceptance when compared to the control cookie. Singh et al. (2015) also reported that the addition of A. *platensis* (>7% incorporation level) to cookies prepared from sorghum and whole wheat flour adversely affected the textural and sensory attributes. In contrast, Abd El‐Baky et al. (2015) found that cookies supplemented with different levels of A. *platensis* biomass (0.3%, 0.6%, and 0.9% incorporation levels) were significantly acceptable for color, odor, flavor, texture, and global appreciation.

### 3.3 Physicochemical tests of juices

Organoleptically optimal samples, control samples (without adding wheat germ powder and spirulina algae), and several other samples were selected to measure other physicochemical properties of juices (Table 3). Tests such as Brix, acidity, pH, formalin index, protein, and dry matter were performed for these samples.

**TABLE 3:** The formulation of selected treatments for screening stage

Types of juices	Wheat germ content (%)	Spirulina content (%)	Total protein content (%)
WGSP	75	25	2
Control (C)	0	0	0
WP	100	0	2
SP	0	100	2
Optimal sensory evaluation (OP)	66.6	33.3	1
SPWP	25	75	2

The results of physicochemical tests on selected samples showed that the addition of spirulina and wheat germ powder had little effect on pH, acidity, and formalin index but had an effect on Brix, dry matter, and protein (Table 4).

**TABLE 4:** Some physicochemical characteristics of screened samples

Samples	Brix	Acidity	Formalin index	pH	Protein	Dry material
WGSP	14.5 ± 0.15^a^	0.23 ± 0.01^a^	1 ± 0.20^a^	3.2 ± 0.10^a^	0.402 ± 0.15^a^	14.59 ± 0.25^a^
C	14 ± 0.13^b^	0.23 ± 0.02^a^	1.4 ± 0.25^b^	3.19 ± 0.13^a^	0.018 ± 0.01^b^	14.26 ± 0.20^a^
WP	13.9 ± 0.1^b^	0.22 ± 0.02^a^	2 ± 0.25^c^	3.37 ± 0.12^b^	0.214 ± 0.07^c^	15.12 ± 0.22^b^
SP	14 ± 0.12^b^	0.22 ± 0.01^a^	1 ± 0.15^a^	3.25 ± 0.10^ab^	0.784 ± 0.11^d^	14.85 ± 0.15^ab^
OP	13.7 ± 0.1^c^	0.22 ± 0.02^a^	1.4 ± 0.2^b^	3.37 ± 0.15^ab^	0.111 ± 0.05^e^	14.46 ± 0.10^a^
SPWP	13.6 ± 0.09^c^	0.22 ± 0.02^a^	1 ± 0.15^a^	3.43 ± 0.17^b^	0.506 ± 0.09^a^	14.83 ± 0.13^ab^

*Note*: Similar letters show no significant difference between means of treatments (*α* = 0.05).

### 3.4 Total phenols, flavonoids, and antioxidant capacity

Phenolic compounds, synthesized as secondary metabolites by plants, but also by cyanobacteria, are considered one of the most important classes of natural antioxidants (El‐Baky et al., 2009). Phenolics, which include simple phenols, flavonoids, phenylpropanoids, tannins, lignins, phenolic acids, and their derivatives, are receiving increasing interest from food manufacturers and consumers for their health benefits (Machu et al., 2015).

**FIGURE 1:**
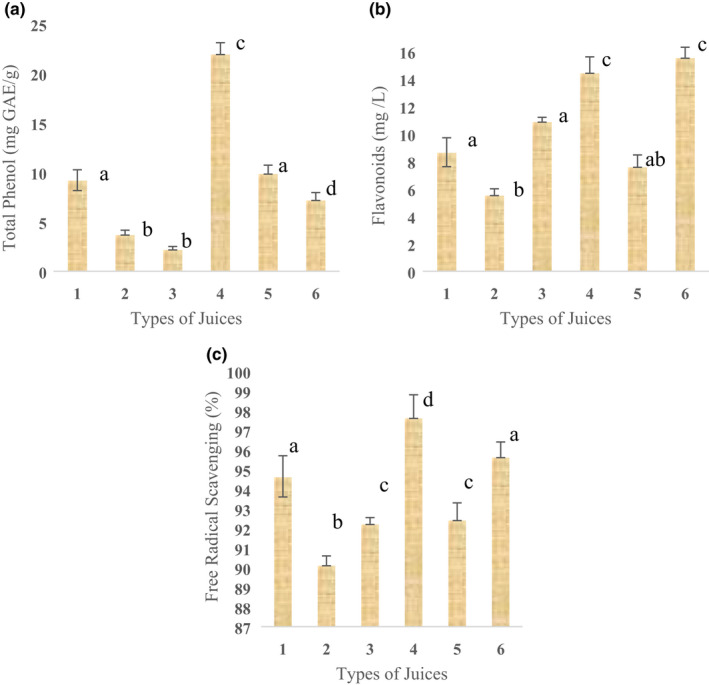
Effect of fruit juice formulation on total phenol (a), flavonoids (b), and free radical scavenging capacity (c): 1: WPSP, 2: C, 3: WP, 4: SP, 5: OP, 6: SPWP. Similar letters show no significant difference between means of treatments (*α* = 0.05)

The results of tests of total phenol, flavonoids, and antioxidant capacity in this study showed that the addition of spirulina and wheat germ powder and especially spirulina had a significant effect on these responses so that the highest values for all three responses in samples containing the maximum amounts of spirulina have been observed (Figure 1). The total phenol content, flavonoids content, and free radical scavenging capacity increased from 3.65 ± 0.5–21.9 ± 1.2 GAE/g, 5.5 ± 0.9–14.4 ± 1.3 mg/L, 90.1 ± 1–97.6 ± 1.3%, respectively, in sample 4 (all 3% protein provided by spirulina) compared to control. Meanwhile, sample 5 as the optimal sample of sensory evaluation had high values of all three responses of total phenol (9.82 ± 0.9 GEA/g), flavonoids (7.56 ± 0.8 mg/L), and antioxidant capacity (92.4 ± 1%), which is very important in term of industrial application of this product.

In 2019, Nikolai et al. related the effects of spirulina on the increase of phenolic substances and antioxidant properties to the presence of a blue pigment called phycocyanin. As shown in Figure 1, there is a linear relationship between the measured responses (total phenol, flavonoids, and antioxidant capacity). This result was confirmed by Siriwardhana et al. (2003) who reported a high correlation between radical scavenging capacity and total phenolic content. Singh et al. (2015) also found a linear positive correlation between A. *platensis* concentration (from 1.6% to 8.4%) in biscuits and antioxidant activity.

There are many studies on the effect of spirulina on these responses in different foods. Niccolai et al. (2019) studied on functional effects of spirulina in crostini (new microalgae‐based sourdough). They reported that the addition of A. *platensis*, with a total phenolic content of 10 mg GAE/g ± 0.1, resulted in a significant (*p* < .05) supplementation of phenolic compounds to the “crostini.” The incorporation of A. *platensis* led to a significant (*p* < .05) increase in the radical‐scavenging capacity for 6 and 10% A. *platensis* “crostini” compared to the control. The control showed a radical scavenging capacity of about 55%, indicating that ingredients in the sourdough preparation already confer a strong antioxidant capacity, which is only slightly increased by A. *platensis* addition. Egea et al. (2014) also reported an increase of about 65% (from 1.4 to 2.3 mg GAE/g) in total phenolic content in 5% A. *platensis* cookies when compared to the control.

As mentioned at the beginning of this section, the addition of wheat germ powder, like spirulina (although with a lower slope), had a significant effect on the nutritional properties measured in this study, especially its antioxidant capacity and flavonoid content. This result is in accordance with the research of Wu et al. (2022) who revealed that the extruded pure wheat bran and heat‐treated wheat germ can effectively increase the antioxidant capacity and the content of the phenolic acid types and flavonoids and they are suitable for producing functional foods.

Also, Zhu et al. (2011) reported that defatted wheat germ (DWG), as a source of natural antioxidants, can be used to formulate nutraceuticals with potential applications to reducing the level of oxidative stress. The antioxidant potency of the DWG extracts could be the basis for its health‐promoting potential.

In another similar research, Majzoobi et al. (2016) evaluated the physicochemical properties of fresh chilled dairy dessert supplemented with wheat germ and concluded that the supplementation of dairy dessert with wheat germ can enhance the nutritional value and antioxidant content of the product.

### 3.5 Mineral measurement

The results show that the addition of spirulina algae and wheat germ powder has significantly affected the amount of most important minerals in the juice (*p* < .05) (Table 5). Wheat germ powder increases iron in the juice. The combination of spirulina algae and wheat germ powder also increased the amount of zinc and calcium compared to other samples. All samples containing spirulina or wheat germ powder or a combination of them had higher levels of magnesium compared to the control sample. Since both spirulina algae and wheat germ powder are important sources of minerals, recording such results are to be expected, and almost the control sample contained the lowest values for all measured minerals.

**TABLE 5:** Some essential mineral content (ppm) in enriched juices with spirulina and wheat germ powder in their different ratios and control samples

Samples	Cu	Fe	Zn	Ca	Mg	K	P
WGSP	0.11 ± 0.01^a^	3.43 ± 0.31^a^	2.24 ± 0.20^a^	50.1 ± 2.2^a^	22.9 ± 0.20^a^	149 ± 3.2^a^	17.1 ± 2.2^a^
C	0.1 ± 0.02^a^	6.78 ± 0.42^b^	0.32 ± 0.08^b^	28.2 ± 1.4^b^	15.3 ± 0.20^a^	109 ± 3.1^b^	5.74 ± 1.3^b^
WP	0.1 ± 0.02^a^	9.77 ± 0.50^c^	0.59 ± 0.10^bc^	47.3 ± 2.1^a^	25.1 ± 0.20^a^	148 ± 4.2^a^	30.7 ± 3.2^c^
SP	0.1 ± 0.02^a^	4.42 ± 0.21^d^	0.78 ± 0.13^c^	32.1 ± 1.8^b^	24.8 ± 0.20^a^	156 ± 4.1^a^	22.7 ± 2.2^d^
OP	0.09 ± 0.01^a^	6.35 ± 0.41^b^	1.49 ± 0.20^d^	35.2 ± 1.7^bc^	19.4 ± 0.20^a^	137 ± 3.30^c^	19.3 ± 1.5^ad^
SPWP	0.11 ± 0.03^a^	6.9 ± 0.53^b^	0.54 ± 0.12^b^	40.9 ± 2.1^d^	26.6 ± 0.20^a^	155 ± 4.20^a^	28 ± 3.2^c^

*Note*: Similar letters show no significant difference between means of treatments (*α* = 0.05).

In confirmation of these results, Saharan and Jood (2017) studied on Vitamins, minerals, protein digestibility, and antioxidant activity of bread enriched with spirulina *platensis* powder and reported that Spirulina fortified bread exhibited the distinctive higher amount of total calcium, phosphorus, magnesium, and iron as compared to control bread. As on increasing the level of Spirulina enrichment resulted in outstanding improvement in mineral contents. Among the enriched bread, 6% Spirulina enriched bread samples had a considerably higher amount than 4% and 2% enriched breads. Also, In vitro availability of calcium, iron, and zinc were recorded higher in all types of enriched bread samples as compared to control samples.

In recent years, various studies have been conducted to enrich juices with various compounds, including plant extracts to increase the content of minerals, vitamins, and antioxidants, which are mentioned below. Tamer et al. (2016) fortified lemonade juice with herbal extract (linden, heather, green tea, lemon verbena, clove, peppermint, ginger, and mate) rich in vitamin C and flavonoids and determined K, Na, Mg, and P values in the lemonades in the range of 178.83–210.98, 33.75–39.13, 22.37–27.89 and 7.22–10.04 mg/kg, respectively. Vignesh et al. (2019) studied on sensory and storage quality of the RTS (ready‐to‐serve) Juice enriched with Papaya leaf flavonoids and concluded that the calcium content in samples with papaya leaf flavonoids was found stable during 45 days of storage. But in samples without papaya leaf flavonoids the calcium content decreased gradually. The phosphorous present in the juice sample decreased as increased in the storage time. The rate of change of phosphorous in both samples with and without papaya leaf flavonoid was the same. The iron content of the juice increased with decrease in the concentration of papaya fruit pulp according to the blending ratio. The values of iron were found stable throughout the storage time of 45 days.

### 3.6 Steady shear rheological test

**FIGURE 2:**
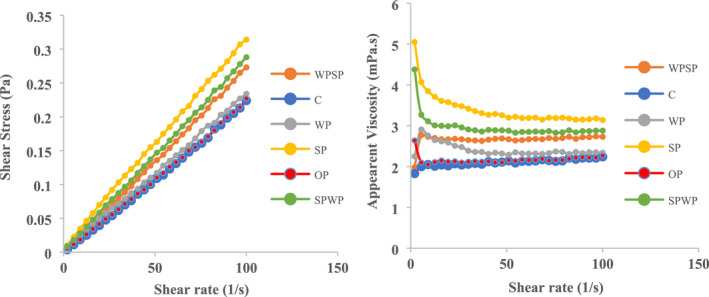
Shear stress and apparent viscosity versus shear rate for the juice samples

The rheological properties of juices are mainly affected by the nature of the juice itself, the presence of polymeric substances such as pectin and gums, fruit pulp content, and the type of thickener agent used. The results of steady shear rheological tests showed that the addition of both spirulina and wheat germ powder, individually and in combination, increased the apparent viscosity, which potentially could increase the desirability of the consumer's mouthfeel (Figure 2). In contrast, Ahmadian Mask et al. (2019) evaluated the effect of wheat germ powder on physicochemical, microbial, and sensory properties of kefir beverage. Their results showed that the samples of kefir beverage containing wheat germ powder showed non‐Newtonian flow behavior according to the power‐law model and the apparent viscosity of the samples significantly decreased with increasing wheat germ concentration. Faccin et al. (2009) formulated a new organic rice bran‐based beverage and evaluated the chemical, sensorial and rheological properties of this product. They reported that the pasteurized rice bran‐based beverage showed the Newtonian fluid behavior.

### 3.7 Dynamic oscillatory shear test

**FIGURE 3:**
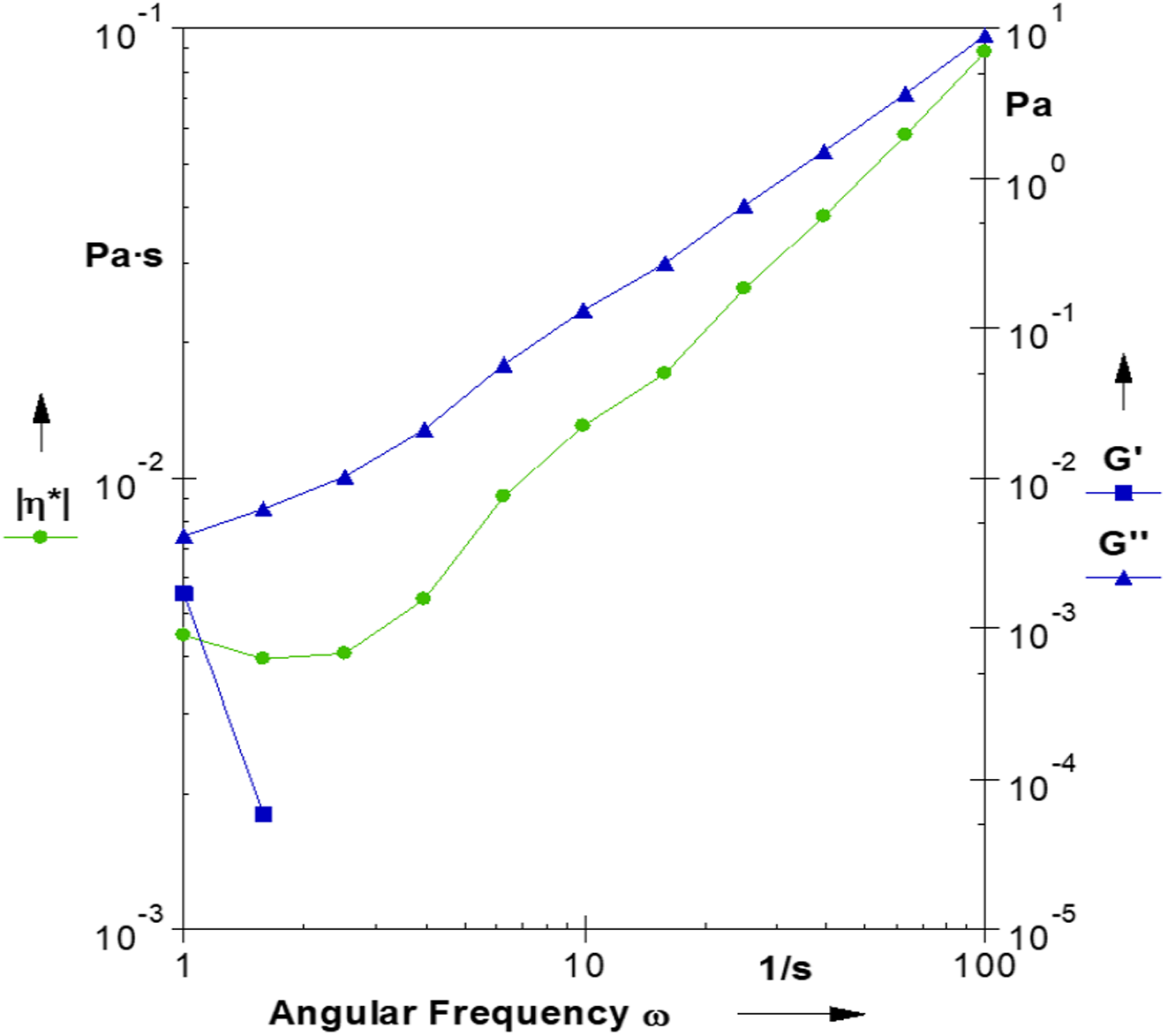
The frequency sweep test of the typical enriched juice samples

Spirulina and wheat germ contain protein and fiber and therefore can affect the rheological properties of fruit juice. Samples containing 2% spirulina (SP) had the highest amount of G′′ and control samples (C) and the optimal sample of sensory evaluation (OP) had the lowest amount of G′′ (Table 6). Figure 3 shows the dynamic mechanical spectra of the typical juice sample as functions of frequency (Hz). The loss modulus (G′′) is predominant for all samples while the storage modulus (G′) had very small amounts in samples containing spirulina and wheat germ powder. Due to the fluidity of all samples, the predominance of the loss modulus (G′′) was observed, and the addition of spirulina algae and wheat germ powder to the samples, at least at low frequencies, has caused to appear the storage modulus (G′).

**TABLE 6:** Rheological characteristics of different enriched juices with spirulina and wheat germ

Sample	Apparent viscosity (cP)	Loss modulus (G′′) (×10^−3^)	Storage modulus (G′) (×10^−3^)	Complex viscosity (η*) (×10^−3^)
WGSP	2.82 ± 0.12^a^	4.4 ± 0.10^a^	1.7 ± 0.14^a^	4.47 ± 0.14^a^
C	2.13 ± 0.10^b^	1.9 ± 0.05^b^	0.1 ± 0.09^b^	3.5 ± 0.1^b^
WP	2.53 ± 0.20^c^	4.01 ± 0.15^a^	2.77 ± 0.1^c^	4.87 ± 0.19^a^
SP	3.03 ± 0.22^a^	7.25 ± 0.12^c^	3.71 ± 0.22^d^	8.15 ± 0.12^c^
OP	2.17 ± 0.15^b^	1.59 ± 0.07^d^	6.19 ± 0.2^e^	1.59 ± 0.06^d^
SPWP	2.66 ± 0.19^c^	4.83 ± 0.19^e^	2.71 ± 0.12^c^	5.54 ± 0.22^e^

*Note*: Loss modulus and Storage modulus, apparent viscosity are reported at angular frequency (1 1/s) and shear rate (50 1/s), respectively.

Similar letters show no significant difference between means of treatments (*α* = 0.05).

This result is consistent with the results of Salinas et al. (2021) when they enriched apple juice with apple fiber and observed a greater increase in the storage modulus (G′) than the loss modulus (G′′) in a frequency sweep test. According to Figure 3, complex viscosity (η*) was also frequency dependent since η* decreased as the frequency increased.

Augusto et al. (2011) reported a change in flow behavior of the products from Newtonian to pseudoplastic and then, Herschel–Bulkley behavior due to fiber addition to the peach juice.

Tizianiand and Vodovotz (Tiziani & Vodovotz, 2005) evaluated the rheological characterization of a novel functional food (tomato juice with soy germ) and they reported that the addition of soy germ increased the viscosity of tomato juice. From the dynamic and steady shear flow experiments, they conclude that the addition of soy germ to tomato juice did not affect qualitatively the rheological properties of tomato juice while the addition of soy protein showed significant qualitative and quantitative differences. Dynamic tests depicted “physical gel” characteristics for all three products. The higher gel strength of the two soy‐containing tomato systems reduced the serum separation and increased the water‐holding capacity of tomato juice. The oscillatory tests of tomato juices showed that the stability and the compatibility between the two different soy ingredients and tomato juice were different. Uribe‐Wandurraga et al. (2021) carried out a study about the physicochemical, rheological, and textural properties of a microalgae‐enriched jam and acclaimed that Jams containing microalgae biomass‐extract showed higher G′, G″, G*, and η* values than their corresponding control samples.

### 3.8 Fatty acid composition determination

**FIGURE 4:**
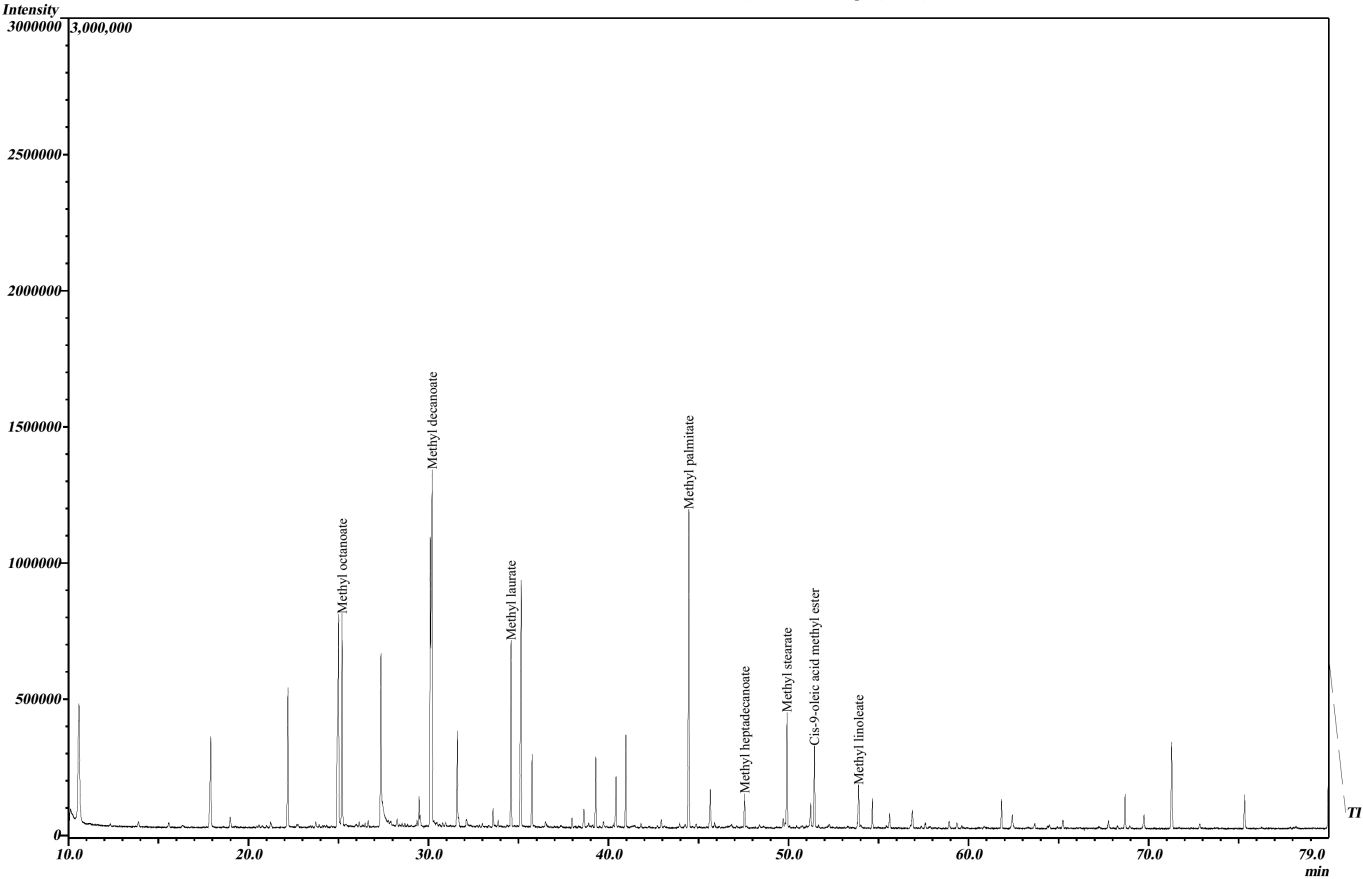
The chromatogram of fatty acid profile of optimal juice sample

The development of cardiovascular diseases, particularly atherosclerosis, is associated with endothelial dysfunction. Saturated fatty acids (SFA) are known to impair, whereas long‐chain n3‐polyunsaturated fatty acids (PUFA) improve endothelial function (Graf et al., 2013). Chromatogram peaks show that the addition of spirulina and wheat germ powder increased the fat, especially long‐chain ω6 and ω9‐ PUFA (linoleic acid and oleic acid) in the juice (Figure 4).

These results are consistent with the fatty acid profiles found in spirulina, which are described below. The fatty acid profile of the spirulina demonstrates that palmitic acid is the most abundant followed by linolenic and linoleic acids (Colla et al., 2004). Colla et al. (2004) acclaimed that the Spirulina produced under the culture conditions is a potential source of gamma linoleic acid for use as a food additive or in capsule form as a nutritional supplement.

Heptadecanoic acid, is rarely found in wheat lipids (Khan, 2016). Among these fatty acids, the most promising one is heptadecanoic acid, which acts against the skin cancer protein (Hsp90) with an effect that is superior to that of the standard drug, dyclonine (Kathiresan, 2020). This special fatty acid, which is present in small amounts in wheat, confirms the presence of wheat bran powder in juice. Another special fatty acid found in chromatogram peaks is decanoic acid. It should be noted that decanoic acid has been introduced to provide seizure control in vivo, yet its mechanism of action remains unclear. Decanoic acid acts as a noncompetitive antagonist at therapeutically relevant concentrations, in a voltage‐ and subunit‐dependent manner and this is sufficient to explain its antiseizure effects (Chang et al., 2016).

### 3.9 Microbial tests

Tests related to the total count, mold, yeast, and E. coli were performed for pasteurized juice samples and the test result was negative for mold, yeast, and E. coli and the total count was less than 1 CFU/ml.

## 4 CONCLUSION

Despite protein's documented health benefits, the act of incorporating protein sources into a beverage usually presents an array of challenges. Proteins often impart “off” flavors or aftertastes, making it necessary for beverage manufacturers to explore masking ingredients. Using of cloudy fruit juices for enrichment by protein sources can decrease these problems. The present study showed that the addition of spirulina algae and wheat germ powder to pear‐cantaloupe juice can significantly enhance the nutritional value of the product in terms not only protein, but also in terms of mineral content and essential fatty acids. Also, the addition of wheat germ powder can change the rheological properties and cause a more desirable mouthfeel. It should be noted that sensory evaluation showed that the best organoleptic samples were those containing lower amounts of spirulina and wheat germ powder and adding these ingredients to fruit juices, especially spirulina algae, should be used with caution. High concentrations of spirulina can cause unpleasant taste, especially after pasteurization, and this can be intensified during storage.

## Data Availability

The data that support the findings of this study are available on request from the corresponding author. The data are not publicly available due to privacy or ethical restrictions.
